# Effects of Computerized Decision Support Systems on Practitioner Performance and Patient Outcomes: Systematic Review

**DOI:** 10.2196/17283

**Published:** 2020-08-11

**Authors:** Clemens Scott Kruse, Nolan Ehrbar

**Affiliations:** 1 School of Health Administration Texas State University San Marcos, TX United States

**Keywords:** CDSS, performance, outcomes

## Abstract

**Background:**

Computerized decision support systems (CDSSs) are software programs that support the decision making of practitioners and other staff. Other reviews have analyzed the relationship between CDSSs, practitioner performance, and patient outcomes. These reviews reported positive practitioner performance in over half the articles analyzed, but very little information was found for patient outcomes.

**Objective:**

The purpose of this review was to analyze the relationship between CDSSs, practitioner performance, and patient medical outcomes. PubMed, CINAHL, Embase, Web of Science, and Cochrane databases were queried.

**Methods:**

Articles were chosen based on year published (last 10 years), high quality, peer-reviewed sources, and discussion of the relationship between the use of CDSS as an intervention and links to practitioner performance or patient outcomes. Reviewers used an Excel spreadsheet (Microsoft Corporation) to collect information on the relationship between CDSSs and practitioner performance or patient outcomes. Reviewers also collected observations of participants, intervention, comparison with control group, outcomes, and study design (PICOS) along with those showing implicit bias. Articles were analyzed by multiple reviewers following the Kruse protocol for systematic reviews. Data were organized into multiple tables for analysis and reporting.

**Results:**

Themes were identified for both practitioner performance (n=38) and medical outcomes (n=36). A total of 66% (25/38) of articles had occurrences of positive practitioner performance, 13% (5/38) found no difference in practitioner performance, and 21% (8/38) did not report or discuss practitioner performance. Zero articles reported negative practitioner performance. A total of 61% (22/36) of articles had occurrences of positive patient medical outcomes, 8% (3/36) found no statistically significant difference in medical outcomes between intervention and control groups, and 31% (11/36) did not report or discuss medical outcomes. Zero articles found negative patient medical outcomes attributed to using CDSSs.

**Conclusions:**

Results of this review are commensurate with previous reviews with similar objectives, but unlike these reviews we found a high level of reporting of positive effects on patient medical outcomes.

## Introduction

### Rationale

Computerized decision support systems (CDSSs) are software programs that support the decision making of patients, practitioners, and staff with knowledge and person-specific information. CDSSs present several tools and alerts to enhance the decision-making process within the clinical workflow [1]. Knowledge-based CDSSs were the earliest classes of CDSSs using a data repository to draw conclusions. Knowledge-based systems use traditional computing methods giving programmed results. Non–knowledge-based CDSSs are the most common forms used today. These systems use artificial intelligence (AI) assistance to augment clinical decisions made at the point of care. AI-supported CDSSs use patient data to analyze relationships between symptoms, treatments, and patient outcomes to make clinical decisions. These patient data are usually derived from electronic health records (EHRs): digital forms of patient records that include patient information such as personal contact information, patient’s medical history, allergies, test results, and treatment plan [2]. Artificial intelligence, software, or algorithms able to perform tasks that normally require human intelligence are integrated into CDSS processes. Data mining, a process usually assisted by AI, is often used by CDSSs to identify new data patterns from large data sets (like patient EHRs) [3]. The conclusions reached by AI used for data mining can be used by both non–knowledge-based CDSSs and knowledge-based CDSSs [3]. CDSSs are integrated into technologies such as computerized physician order entry (CPOE) [4] tools and electronic medical record (EMR)/EHR databases and use a wide variety of drug, patient, and treatment data and more to make clinical decisions that provide the best recommendations for treatment. CDSS utility varies widely, drawing conclusions about different ailments, disorders, and syndromes. Prospects for this technology may employ patient preferences or financial capabilities.

In prior studies, CDSSs have been shown to improve practitioner performance, but the effects on patient outcomes were inconsistent and required further study. A review conducted in 1998 evaluated studies for the previous 5 years and found a benefit to physician performance in 66% of studies analyzed (n=65), but only 14 of those analyzed discussed outcomes, so no conclusions were made [5]. The review was repeated in 2005 with a larger sample (n=100) and found a positive impact on physician performance in 64% of studies analyzed, but like the 1998 review, effects on patient outcomes were insufficient to make generalizations [6]. In 2010, a research protocol was registered to repeat the review, but no publication followed. In 2011, the review was repeated with a similar size of articles analyzed (n=91) and identified a positive effect of CDSSs on practitioner performance for 57% of articles analyzed; however, consistent with previous reviews, no conclusions could be made concerning patient outcomes [7].

Since the last publication on this topic in 2011, CDSSs have seen significant industry growth, becoming more accessible, cost-effective, and reliable and possessing greater computational power [8]. In addition to hardware improvements, the inclusion of software such as artificial intelligence (AI) programs is growing rapidly in CDSSs, but as of yet these improvements have not been systematically reviewed to determine any impacts they might have on patient outcomes and practitioner performance.

### Objective

The purpose of this systematic review is to conduct a similar review to those from 1998 and 2005 to analyze the association between CDSSs, practitioner performance, and patient outcomes. The methods used in the 2010 manuscript were never published, and those used in the 2011 review were significantly different than those in 1998 and 2005. The taxonomy of CDSSs has changed greatly since 1998, so search terms used 23 years ago will not be relevant today. CDSS employment is rapidly growing, especially with increased access to CDSS AI-supported software. Because the effects are understudied, our goal is to review the effectiveness of CDSS technologies, their employment, and their overall utility.

## Methods

### Protocol Registration and Eligibility Criteria

This review was not registered. The methods followed a technique of sharing workload from the Assessment of Multiple Systematic Reviews (AMSTAR) [9]. The format of the review uses the Preferred Reporting Items for Systematic Reviews and Meta-Analyses (PRISMA) [10]. Conceptualization of the overall review, including standardized data extraction tools, follows the Kruse protocol for writing systematic reviews in a health-related program [11]. Articles were eligible for inclusion if they were published in the English language within the last 10 years, had full text available, and reported on the elements of the objective statement: measures of effectiveness of CDSSs on practitioner performance or patient outcomes. A 10-year window was justified because we wanted the research to be current, and this exceeds the window of the 1998 and 2005 reviews, which used only 5 years. At first, we limited the search to studies in peer-reviewed journals, but because our sample was too small, we expanded the search to include grey literature. However, we limited our choices to use only those that had results.

### Information Sources

Five common research databases were queried: PubMed (the web-based components of MEDLINE, life science journals, and online books), CINAHL, Embase, Web of Science, and Cochrane (reviews, controlled trials, methodologies, and health technology assessments). Searches were conducted from January 29 to January 31, 2020. Databases were chosen at the recommendation of the National Institutes of Health, which recommends at least three databases: PubMed, Embase, and Cochrane [12]. This practice also follows established practice in published systematic reviews [11].

### Search and Study Selection

Searches in each database were identical: (“Clinical decision support systems” OR “computerized provider order entry” OR “diagnosis, computer assisted” OR “drug therapy, computer-assisted” OR “expert systems”) AND (“patient reported outcomes” OR “practitioner performance”). Embase and Web of Science do not allow Boolean searches, so an advanced search was used. Articles were eligible for inclusion if they were published in the last 10 years and discussed both CDSSs and either practitioner performance or patient-reported outcomes. We excluded reviews. In CINAHL, we excluded MEDLINE to avoid duplication with the results from PubMed.

The search strings for the 1998 and 2005 reviews were not available, but the search string for the 2011 study was available: (literature review[tiab] OR critical appraisal[tiab] OR meta analysis[pt] OR systematic review[tw] OR medline[tw]) AND (medical order entry systems[mh] OR medical order entry system*[tiab] OR computerized order entry[tiab] OR computerized prescriber order entry[tiab] OR computerized provider order entry[tiab] OR computerized physician order entry[tiab] OR electronic order entry[tiab] OR electronic prescribing[mh] OR electronic prescribing[tiab] OR cpoe[tiab] OR drug therapy, computer assisted[mh] OR computer assisted drug therapy[tiab] OR decision support systems, clinical[mh] OR decision support system*[tiab] OR reminder system*[tiab] OR decision making, computer assisted[mh] OR computer assisted decision making [tiab] OR diagnosis, computer assisted[mh] OR computer assisted diagnosis[tiab] OR therapy, computer assisted[mh] OR computer assisted therapy[tiab] OR expert systems[mh] OR expert system*[tiab]). It is important to note the limited terms used for CDSSs also included lesser known terms indexed by PubMed’s Medical Subject Headings: clinical decision support; clinical decision supports; decision support, clinical; support, clinical decision; supports, clinical decision; decision support, clinical; and decision support systems, clinical. Searching for CPOE also included order entry systems, medical; medication alert systems; alert system, medication; medication alert system; system, medication alert; alert systems, medication; computerized physician order entry system; CPOE; computerized provider order entry; and computerized physician order entry. Searching for diagnosis, computer assisted also included the following: computer-assisted diagnosis; computer assisted diagnosis; computer-assisted diagnoses; and diagnoses, computer assisted. Searching for drug therapy included the following: drug therapy, computer assisted; therapy, computer-assisted drug; computer-assisted drug therapies; drug therapies, computer-assisted; therapies, computer-assisted drug; therapy, computer assisted drug; computer-assisted drug therapy; computer assisted drug therapy; protocol drug therapy, computer-assisted; and protocol drug therapy, computer assisted. A search of expert systems also included expert system; system, expert; and systems, expert.

Abstracts were independently screened by each reviewer, and a consensus meeting was called to discuss disagreement. A kappa score was calculated to provide a measure of agreement between reviewers.

### Data Collection and Data Items

A standardized Excel spreadsheet (Microsoft Corporation) was used as a data extraction tool, in accordance with the Kruse protocol [11]. This tool acted as a template for reviewers to collect study design, participants, sample size, intervention, observed bias, and effect size, where applicable. A literature matrix was created to list and organize all articles, extract data between multiple reviewers, and discuss observations in consensus meetings. Three consensus meetings were held for reviewers to discuss disagreement and share observations. This practice created a synergy effect and ensured everyone progressed with a like mind.

### Risk of Bias in Individual Studies

Reviewers noted any observation of bias. We used the Johns Hopkins Nursing Evidence-Based Practice (JHNEBP) tool as a quality assessment of studies analyzed. Other forms of bias were noted as well, which are described in risk of bias across studies.

### Synthesis of Results

The Excel spreadsheet was used to synthesize our observations and data collected. The spreadsheet enabled a narrative analysis which identified themes, as is the practice in multiple disciplines. We did not combine results of studies because this was not a meta-analysis.

### Risk of Bias Across Studies

Additional forms of bias other than selection bias were noted on the spreadsheet such as localized studies or surveillance bias.

### Additional Analysis

Reviewers read each article two times [11]. During the second reading, reviewers made independent notes of major themes related to the objective, using the Excel data extraction tool. After a third consensus meeting debriefing the observations and themes, detailed notes were formulated about health policy implications of telemedicine. Frequency of occurrence of each of the major common themes was captured in affinity matrices for further analysis. Data and calculations are available upon request.

## Results

### Study Selection and Study Characteristics

**Figure 1:**
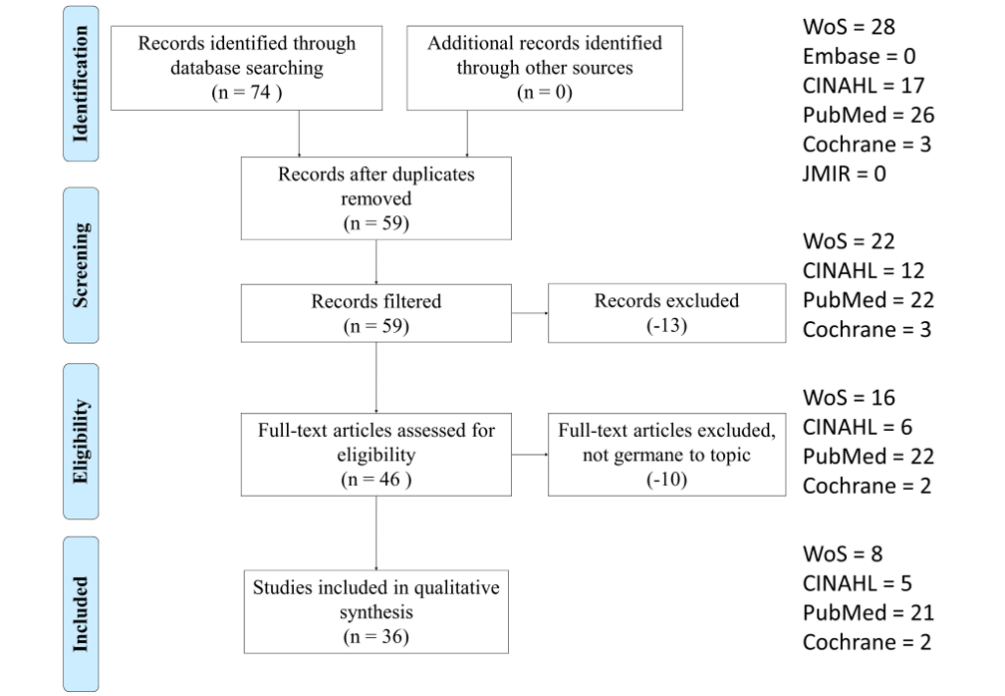
Article selection process with selection criteria.

The study selection process is illustrated in Figure 1. The 74 results from the search string in five databases were placed into an Excel spreadsheet and shared among reviewers for selection and analysis. Filters were applied in each database to capture only the last 10 years (January 30, 2011, to January 30, 2020). Reviewers independently removed duplicates and screened abstracts. A statistic of agreement, kappa, was calculated. The kappa score produced was .98, showing almost complete agreement on all reviewed articles [13,14]. The remaining 36 results were read in full for relevance. Observations for the 36 articles that remained were placed in an Excel spreadsheet for independent data analysis.

Reviewers collected standard patient/participants, intervention, comparison, outcome, study design (PICOS) observations plus indications of either practitioner performance or patient medical outcomes (Multimedia Appendix 1). Bias was also noted. Following the Kruse protocol, observations were distilled into themes for further analysis. Three consensus meetings were used to discuss disagreement. A summary of all observations is listed in Table 1. Articles are listed in reverse chronological order. The details extracted were year of publication, authors, title, study design, participants, sample size, intervention, bias, and observations about barriers or facilitators to the adoption of telemedicine.

### Risk of Bias Within Studies

Bias was not observed in all studies analyzed. A full review of the bias observed is provided in Multimedia Appendix 1. The JHNEBP tool found no quality measure below Level IV or C.

### Results of Individual Studies

General observations and thematic analysis are listed in Table 1. Articles are listed in reverse chronological order. A table of PICOS is provided in Multimedia Appendix 1.

**Table 1:** Summary of analysis.

Authors	Efficiency (practitioner performance)	Efficiency themes	Effectiveness (medical outcomes)	Effectiveness themes
Grout et al [15]	Practitioner performance not discussed	Not reported or discussed	Self-reporting by adolescents increased (doubled) by 19.3 percentage points	Improved screening
Connelly et al [16]	Number of prescriptions written for migraines increased significantly; average length of time per use of tool was 3.3 minutes	More accurate prescribing	Medical outcomes not reported or discussed	Not reported or discussed
Salz et al [17]	Facilitated a more comprehensive visit	Improved care plans	Provided a way for patients to chronicle other physicians who had been involved in medical decisions enabling doctors to communicate	Improved feedback
Kirby et al [18]	Increased referral awareness by providers for patients with severe aortic stenosis (which is a known quality issue); increase in referral rate from 72% to 98%	Increased awareness	Medical outcomes not reported or discussed	Not reported or discussed
Dolan and Veazie [19]	Practitioner performance not statistically different	No difference reported	Medical outcomes not reported or discussed	Not reported or discussed
Jackson and De Cruz [20]	Practitioner performance not discussed	Not reported or discussed	Improvement in relapse duration, medication adherence, cost, and number of clinic visits	Improved symptoms
Caballero-Ruiz et al [21]	Face-to-face visits with patients reduced by 89% time devoted by clinicians to patient evaluation was reduced by 27%; automatic detection of 100% of patients who needed insulin therapy	Improved performance	Diet prescriptions provided without clinician intervention; patients were very pleased with the tool	Improved disease management
Raj et al [22]	System did not improve pain intensity, therefore no significant differences in dose of opiates compared with control; had no effect on practitioner performance	No difference reported	Did not improve or worsen pain management	No difference reported
Mooney et al [23]	Enabled providers to follow up based on feedback from patients	Better follow-up with patients	Intervention group demonstrated fewer severe and moderate symptoms	Improved symptoms
Baypinar et al [24]	Correct prescribing increased from 54% to 91% (*P*<.01) for folic acid and 11% to 40% (*P*<.001) for vitamin D, and stopped orders increased from 3% to 14% (*P*<.002)	More accurate prescribing	Medical outcomes not reported or discussed	Not reported or discussed
Zini et al [25]	Practitioners improved prevention, diagnosis, and treatment	Improved care plans	Medical outcomes not reported or discussed	Not reported or discussed
Muro et al [26]	Practitioner performance not discussed	Not reported or discussed	Improved symptoms; decreased adverse events	Improved symptoms
Kistler et al [27]	Practitioner performance not discussed	Not reported or discussed	More patients agreed to screening in the intervention group than the control	Improved screening
Lawes and Grissinger [28]	Practitioners performed worse when CDSS^a^ was not available or when incorrect data were entered for weight	More accurate prescribing	Adverse drug events no doubt occurred because of error, but no outcomes were discussed	Not reported or discussed
Kouladjian et al [29]	Average time to complete task to recognize sedative and anticholinergic medicines in practice was 7:20 (SD 1:45) minutes	Improved performance	Medical outcomes not reported or discussed	Not reported or discussed
Norton et al [30]	Surgeons rated the tool very useful or moderately useful (25%), neutral (47%), or moderately useless or not useful (28%)	Improved care plans	Medical outcomes not reported or discussed	Not reported or discussed
Pombo et al [31]	Resolving missing data	Improved documentation	Resolving missing data in daily diary improved the feedback loop to the pain manager	Improved feedback
Cox and Pieper [32]	Practitioner performance not discussed	Not reported or discussed	Treatment in the doxazosin arm was stopped early due to a 1.25-fold increase in the incidence of CVD^b^ and a 2-fold increase in the incidence of heart failure compared with the diuretic arm	Improved efficacy
Schneider et al [33]	Once CDSS scored significantly more exams as appropriate; better interface of one CDSS versus the other influenced provider willingness to use the CDS system	Improved screening; improved buy-in of CDSSs	Medical outcomes not reported or discussed	Not reported or discussed
Zhu and Cimino [34]	Accuracy improved: reduced inaccuracy	Improved accuracy and performance	Improved patient safety	Improved safety
Utidjian et al [35]	Proportions of doses administered declined during the baseline seasons (from 72% to 62%) with partial recovery to 68% during the intervention season; palivizumab-focused group improved by 19.2 percentage points in the intervention season compared with the prior baseline season (*P*<.001), while the comprehensive intervention group only improved 5.5 percentage points (*P*=.29); difference in change between study groups was significant (*P*=.05)	More accurate prescribing	A quality improvement initiative supported by CDS and workflow tools integrated in the EHR^c^ improved recognition of eligibility and may have increased palivizumab administration rates; palivizumab-focused group performed significantly better than a comprehensive intervention	Improved disease management
Semler et al [36]	No statistically significant difference in performance (also low use of tool)	No difference reported	No statistically significant difference: mortality 14% versus 15%, ICU^d^-free days 17 versus 19, vasopressor-free days 22.2 versus 22.6	No difference reported
Peiris et al [37]	Patients more likely to receive screening with CDSS (63% vs 53%); no improvements in prescription of recommended medications at the end of the study	Improved screening	Improved cardiovascular disease risk management; no difference in prescription rates	Improved disease management
Chow et al [38]	Only one-quarter of patients received antibiotics despite recommendations of CDSSs	More accurate prescribing	Patients aged <65 years had greater mortality benefit (OR^e^ 0.45, 95% CI 0.20-1.00; *P*=.05) than patients >65 years (OR 1.28, 95% CI 0.91-1.82; *P*=.16); no effect was observed on incidence of *Clostridium difficile* (OR 1.02, 95% CI 0.34-3.01) and multidrug-resistant organism (OR 1.06, 95% CI 0.42-2.71) infections; no increase in infection-related readmission (OR 1.16, 95% CI 0.48-2.79) was found in survivors; receipt of CDSS-recommended antibiotics reduced mortality risk in patients aged ≤65 years and did not increase risk in older patients	No difference reported
Wilson et al [39]	Practitioner performance not discussed	Not reported or discussed	Improved self-efficacy and decreased fecal aversion	Improved efficacy
Loeb et al [40]	Training greatly improved documentation	Improved documentation	Medical outcomes not reported or discussed	Not reported or discussed
Mishuris et al [41]	Practitioner performance not discussed	Not reported or discussed	Patients who visited clinics missing at least one of the CDSS functions were more likely to have controlled blood pressure (86% vs 82%; OR 1.3, 95% CI 1.1-1.5) and more likely to not have adverse drug event visits (99.9% vs 99.8%; OR 3.0, 95% CI 1.3-7.3)	Improved symptoms
Dexheimer et al [42]	No difference in time to disposition decision; no change in hospital admission rate; no difference in ED^f^ length of stay	No difference reported	CDSS supported communication between patient and provider	Improved feedback
Heisler et al [43]	Practitioner performance not discussed	Not reported or discussed	Decrease in diabetes distress, but no difference in other outcomes	Improved symptoms
Eckman et al [44]	Decisions are based on >0.1 QALYs^g^; tool identified the 50% who would benefit from this threshold	Improved performance	Significant gain in quality-adjusted life expectancy	Improved mortality
Zaslansky et al [45]	Audit, feedback, and benchmarking provided to practitioners to identify when their practice is not in line with data	Improved benchmarking	Provides real-time feedback on PROs^h^	Improved feedback
Lobach et al [46]	No treatment-related differences between groups	No difference reported	Among patients <18 years, those in the email group had fewer low severity (7.6 vs 10.6/100 enrollees; *P*<.001) and total ED encounters (18.3 vs 23.5/100 enrollees; *P*<.001) and lower ED ($63 vs $89, *P*=.002) and total medical costs ($1736 vs $2207, *P*=.009); patients who were ≥18 years in the latter group had greater outpatient medical costs	Improved symptoms
Barlow and Krassas [47]	Annual cycle of care plans increased by 12%	Improved care plans	Patients better able to meet targets for microalbumin; glycemic control well managed	Improved symptoms
Robbins et al [48]	A total of 90% of providers involved with the RCT^i^ supported adopting the intervention	Improved buy-in of CDSSs	Increased CD4+ lymphocyte count and reduced suboptimal follow-up appointment	Improved symptoms
Chen et al [49]	New CDSS identified 70 records needing reassessment of triglyceride level	Improved screening	Medical outcomes not discussed	Not reported or discussed
Seow et al [50]	A total of 87% of respondents strongly agreed or somewhat agreed that the “ESAS^j^ was important to complete because it helped the health care team to know what symptoms [they] were having and how severe they were”	Improved screening	A total of 79% of respondents rated that their “pain and other symptoms have been controlled to a comfortable level” always or most of the time compared with 8% of respondents who rated this as rarely or never occurring	Improved symptoms

^a^CDSS: computerized decision support system.

^b^CVD: cardiovascular disease.

^c^EHR: electronic health record.

^d^ICU: intensive care unit.

^e^OR: odds ratio.

^f^ED: emergency department.

^g^QALY: quality-adjusted life year.

^h^PRO: patient-reported outcome.

^i^RCT: randomized controlled trial.

^j^ESAS: Edmonton Symptom Assessment System.

### Risk of Bias Across Studies

Multimedia Appendix 1 provides a table of PICOS and bias. Outcomes are reported in Table 1. Bias was similar across articles reviewed: most research took place in one facility, organization, or state, which is a form of selection bias and limits the broad application of results. A sample taken from a limited geographic area is inherently limited in its ability to generalize results to the general population unless steps have been taken to ensure the sample is representative of the population.

### Additional Analysis

Twelve themes were identified for practitioner performance, two of which were no difference and not discussed. These themes are listed in Table 2 in order of occurrence first for positive effect followed by no difference and not discussed.

**Table 2:** Summary of themes identified for practitioner performance (n=38).

Efficiency themes	Occurences	Incidence, n (%)
More accurate prescribing	16,24,28,35,38	5 (13)
Improved screening	33,37,49,50	4 (11)
Improved performance	21,29,34,44	4 (11)
Improved care plans	17,25,30,47	4 (11)
Improved documentation	31,40	2 (5)
Improved buy-in of CDSSs^a^	33,48	2 (5)
Increased awareness	18	1 (3)
Better follow-up with patients	23	1 (3)
Improved accuracy	34	1 (3)
Improved benchmarking	45	1 (3)
No difference reported	19,22,36,42,46	5 (13)
Not reported or discussed	15,20,26,27,32,39,41,43	8 (21)

^a^CDSS: computerized decision support system.

As illustrated, 66% (25/38) of the occurrences of themes identified 10 positive indicators of practitioner performance [16-18,21,23-25,28-31,33-35,37,38,40,44,45,47-50]. Practitioner performance was reported as more accurate prescribing, improved screening of patients, improved overall performance, increased awareness of patient conditions, improved follow-up due to better communication with patients, improved accuracy of diagnosis, improved documentation, improved benchmarking, improved care plans, and improved buy-in of CDSSs. A total of 21% (8/38) of articles did not discuss practitioner performance [15,20,26,27,32,39,41,43].

Practitioners using CDSSs experienced more accurate prescribing [16,24,28,35,38], improved screening [33,37,49,50], improved overall performance [21,29,34,44], improved care plans [17,25,30,47], improved documentation [31,40], overall improved buy-in for CDSSs [33,48], increased awareness of needs of patients [18], improved follow-up with patients due to enhanced communication channels enabled by the application [23], improved accuracy of diagnosis [34], and improved benchmarking [45].

Nine themes were identified for patient medical outcomes, two of which were no difference and not discussed. These themes are listed in Table 3 by order of greatest occurrence for positive effect followed by no difference and not discussed.

**Table 3:** Summary of themes identified for patient medical outcomes (n=36).

Effectiveness themes	Occurences	Incidence, n (%)
Improved symptoms	20,23,26,41,43,46-48,50	9 (25)
Improved feedback	17,31,42,45	4 (11)
Improved disease management	21,35,37	3 (8)
Improved efficacy	32,39	2 (6)
Improved screening	15,27	2 (6)
Improved safety	34	1 (3)
Improved mortality	44	1 (3)
No difference reported	22,36,38	3 (8)
Not reported or discussed	16,18,19,24,25,28-30,33,40,49	11 (31)

As illustrated, 61% (22/36) of occurrences of themes identified 7 positive patient medical outcomes as a result of using CDSSs [15,17,20,21,23,26,27,31,32,34,35,37,39,41-48,50]. Patients experienced improved symptoms [20,23,26,41,43,46-48,50], improved feedback from provider [17,31,42,45], improved disease management [21,35,37], improved efficacy of treatment [32,39], improved screening [15,27], and improved safety [34], and one study even reported improved mortality [44]. Although 11 articles did not discuss patient medical outcomes [16,18,19,24,25,28-30,33,40,49], only 3 reported no statistically significant difference in outcomes between control and intervention groups [22,36,38].

## Discussion

### Summary of Evidence

Our review methodology enabled a meticulous evaluation of the efficiency and effectiveness of CDSSs for practitioner performance and medical outcomes. A summary of the findings from the review are listed in Table 1. Of the 36 articles analyzed that reported efficiency or effectiveness, 25 reported positive performance and 22 reported positive outcomes; 9 did not report practitioner performance and 11 did not report patient medical outcomes.

Commensurate with previous reviews on this topic [6,7], a majority of articles analyzed reported improvement in practitioner performance [16-18,21,23-25,28-31,33-35,37, 38,40,44,45,47-50], but contrary to the previous reviews, our review found articles that reported patient outcomes, and a majority were positive outcomes [15,17,20,21,23,26,27,31,32,34,35,37,39,41-48,50]. Although 9 articles did not discuss practitioner performance [15,20,26,27,32,39,41,43], only 5 articles reported no difference in productivity [19,22,36,42,46].

The decision of whether to adopt a CDSS is one of complexity and change management. Providers and administrators need to discuss the advantages and disadvantages. The organization’s infrastructure must support the application, providers must be trained on how to implement it, and administrators must ensure that budget and organizational dynamics can afford acquisition and implementation. The literature is clear in the efficacy of CDSSs, and this should assist organizations in gaining user acceptance. Providers should carefully integrate CDSSs into their processes and clinical practice guidelines to ensure they are an asset more than a hindrance. They should be used to augment patient care rather than coming between patients and providers.

It is interesting that previous reviews did not find results of medical outcomes. This could have been a limitation in search strategy. It could also be due to the maturation of CDSSs in general. At the time the other reviews were conducted, it may have just been too soon for reviews to see the positive results in medical outcomes.

Because CDSSs present providers with knowledge-based information at the point of care, they augment decision making. Timely tools are available to providers through CDSSs that may not otherwise be available at the point of care. AI-supported recommendations provided by CDSSs analyze symptoms, possible treatments, clinical practice guidelines, and patient outcomes [1,2]. These capabilities are most likely the catalyst for improved practitioner performance and patient outcomes.

There does not appear to be one CDSS panacea for all practices, specialties, or templates. The literature is mixed on which products are best of breed systems. Clearly, additional research should continue to be conducted in this valuable area of medical practice. While other industries have fully embraced the digitized environment, health care in general has been slow to adopt, which is understandable when health is at stake. Based on the results of this review compared with similar ones in the past, CDSSs are diffusing across the health care industry as the systems improve. Further research into CDSSs should look to improve productivity and standardize their integration into clinical practice guidelines.

Another interesting note is that alert fatigue was not raised in any of the studies analyzed. Alert fatigue is a known phenomenon and worthy of note [51]. It is attributed to medical error in the areas of pharmacy and physician ordering systems, which are common attributes in CDSSs [52]. Even in clinical trials, alert fatigue is known to be persistent over time [53]. It is interesting that it was not noted, and if it was not noted, it was not controlled for in the studies analyzed.

### Limitations

The small group of articles for analysis was a limitation. Only 36 articles met the selection criteria. A larger group for analysis would strengthen the external validity of the results because we could be better assured that our group is representative of the population. The effects of selection bias were reduced using multiple reviewers to screen and analyze articles [9]. Only two reviewers screened abstracts and analyzed articles for themes. One additional reviewer might have increased the number of observations. Publication bias was reduced through the inclusion of grey literature that included more than just peer-reviewed material; however, these articles were discarded if they did not include results. We considered only articles published in the English language. It is possible that additional observations could have been gained by expanding the search to other languages. This review is also limited by the techniques used in the trials analyzed, and statistics and effect sizes could not be combined due to the wide range used in the articles. We analyzed both qualitative and quantitative methods, and effect size is only viable for the latter. Sample sizes were widely different between studies analyzed, ranging from 6 to 900 million. Such a wide disparity makes consolidation of results difficult. We also did not analyze or compare the heuristics and algorithms used by CDSSs within the studies. To compensate for a limitation from a similar review in 2005, we expanded our analysis beyond randomized controlled trials to pre-post and other designs [6].

### Conclusion

Overall , the research generally supports the efficiency of CDSS technologies for practitioner performance [16-18,21,23-25,28-31,33-35,37,38,40,44,45,47-50] and effectiveness in patient medical outcomes [15,17,20,21,23,26,27,31,32,34,35,37,39,41-48,50]; however, a further in-depth review of their effectiveness, in particular for aspects such as the avoidance of alert fatigue and extension of CDSS utility, is important. Decision-support tools extend beyond the practitioner to the patient, and some tools are not software-based but based on patient-reported data [46]. The implementation of CDSSs can mutually benefit the practitioner and patient, and they show great promise for health care in the future.

## Appendix

Multimedia Appendix 1 Participants, intervention, comparison, outcome, study design table.

## Abbreviations

AI: artificial intelligence
AMSTAR: Assessment of Multiple Systematic Reviews
CDSS: computerized decision support system
CPOE: computerized physician order entry
EHR: electronic health record
EMR: electronic medical record
JHNEBP: Johns Hopkins Nursing Evidence-Based Practice
PICOS: patient/participants, intervention, comparison, outcome, study design
PRISMA: Preferred Reporting Items for Systematic Reviews and Meta-Analyses
