# Supplementary material for: Effects of Computerized Decision Support Systems on Practitioner Performance and Patient Outcomes: Systematic Review
Source: JMIR Med Inform. 2020 Aug 11;8(8):e17283. doi: 10.2196/17283 (PMC7448176; doi:10.2196/17283)
Supplement: Multimedia Appendix 1 [file medinform_v8i8e17283_app1.docx]

**Appendix A: PICOS+bias**

| Authors_ | Subjects / participants | Technology intervention | Study design | Sample size | Statistics used | Effect size | Bias |
| --- | --- | --- | --- | --- | --- | --- | --- |
| Grout et al [15] | Adolescents 12-18 yo (mean age 14), (1066 control, 1365 intervention), 48% female 52% male | 2 electronic workflow alerts | Interrupted time-series, interventional design | 2670 pre-visit screenings across 19 preintervention, 7 intervention, and 44 postintervention weeks | CI 95%, t-test | not discussed | Adolescents only, which limits generalization |
| Connelly et al [16] | Primary care pediatricians and other family medicine physicians and nurse practitioners | Online clincial decision support tool | Prospective observational study | 53 PCPs and 50 FMPs/NPs | t-test (comparison of means) (P<.1) | not discussed | Not identified |
| Salz et al [17] | Adults who recovered from head and neck cancer (n=10) and their providers (n=10) | Head and Neck Cancer Survivorship Tool: Assessment and Recommendation (HNC-STAR) | Observational | 10 Nurse Practitioners 10 cancer survivors | not discussed | not discussed | Not identified |
| Kirby et al [18] | Providers at 13 hospitals in the Midwest that implemented EpicCare and their patients | CDSS for Cardiology and primary care | Pre-post | 1800 physicians, 800 advanced practice providers, 34,561 transthroacic echocardiograms, 611 referrals | *t*-test (comparison of means) (*P*<.001) | not discussed | Convenience sample of existing providers at 3 hospitals in Midwest |
| Dolan & Veazie [19] | Survey respondents, 35 and older, demographically representative of the U.S., for whom cardiology prevention is recommended | Multifactor decision making tool for patients | Survey (retrospective) and qualitative | 636 | Descriptives, Chi Squared, and Kruskal Wallis | not discussed | Only those who responded to the survey from the survey pool were eligible to participate |
| Jackson & De Cruz [20] | Patients in Melbourne Australia | CDS for Inflamatory Bowel Disease management | Focus group with qualitative analysis | 31 (11 physicians, 6 nurses) | qualitative analysis | not discussed | small sample size inhibits external validity. Limited sample at one organization also limits external validity |
| Caballero-Ruiz et al [21] | Adults with gestational diabetes | Web based telemedicine platform for gestational diabetes | Observational | 90 patients | Mixed methods | not discussed | Telemedicine only. A tool for face-to-face was not evaluated |
| Raj et al [22] | Adults, pain management patients (control, n=80; intervention, n=134) | Combat system to assess pain manamagement at point of care | Controlled before and after study | 214 | Mixed methods | not discussed | Not identified |
| Mooney et al [23] | chemo patients in U.S. | Symptom Care at Home vs. usual care | Longitudinal Randomized Control Trial | 358 | Mixed effects linear modeling and negative binomial regressions. Independent *t*-tests and Chi squared | 80% power, effect size of 0.50 with alpha of 0.05 | Only patients at one facility were eligible to participate |
| Baypinar et al [24] | providers prescribing Methotrexate without folic acid, biophosphonate without vitamin D, or sodium lowering drug prescribed for patient with sodium levels < 130 mmol/l, in one hospital in the Netherlands | Three algorithms in CDSS providing alerts during prescribing process | Pre-post | 1031 orders | Chi squared | not discussed | only providers at one hospital in the Netherlands were eligible to particpate. Alerts based on Netherlands clinical practice guidelines for prescribing. |
| Zini et al [25] | healthy volunteers at one organization | HL7 Virtual Medical Record for Clinical Decision Support Logical Model, Release 2. It includes 1) education, 2) suggestions for prevention, 3) diet and habit suggestions, 4) medication reminders, 5) functional assessment, 6) sensor interface, 7) integration with EHR | Delphi | 15 | qualitative analysis | not discussed | small sample size inhibits external validity. Limited sample at one organization also limits external validity |
| Muro et al [26] | none | DESIREE project | Delphi | 0 | not discussed | not discussed | Development of DESIREE based on research and some feedback.  Development of new CPGs based on non-compliance might be problematic. |
| Kistler et al [27] | English-speaking primary care patients aged 70–84 years in 14 practices in central North Carolina | Colorectal decision aid for patients | Randomized Control Trial | 424 | not discussed | not discussed | North Carolina (U.S.), primary care, part of 14 clinics in the Duke University Health System only, which limits external validity |
| Lawes & Grissinger [28] | Medication errors reported in 2nd and 4th quarters of the | CDS | Secondary data analysis | 671 | qualitative analysis | not discussed | Reports reported only in one state in the U.S. which limits external validity |
| Kouladjian et al [29] | Adult pharmacists | Drug burden index calculator | Case study | 10 | Cohen's Kappa | not discussed | Selection bias (most of the participants considered themselves tech saavy) |
| Norton et al [30] | Adult surgeons operating on 1,006 patients | Decision support for safer surgery (DS3) | Qualitative | 23 | Summary statistics | not discussed | surgeons in Alabama and Utah only |
| Pombo et al [31] | Adults who were surgery patients, ages ranged from 18-75 | Using regression model to resolve missing data in pain management | Quasi experimental | 31 | Kruskal-Wallis and Tukey-Kramer | not discussed | Conducted in a surgery department in Portugal only |
| Cox & PieperL [32] | Patients within national system of Nova Scotia, Canada | Comparative Effectiveness Research on existing data | Secondary data analysis | 42,418 | Stratification and matching, regression and propensity scoring | not discussed | Data collected in Nova Scotia, Canada. Limited data collection area limits external validity |
| Schneider et al [33] | MRI and CT orders | Medicalis SmartReq and Nuance RadPort CDS systems | Retrospective observational | 2,000 | Logistic regression | not discussed | Data collected during over two periods of three months each. Such short periods limit the broad applicability of the results. |
|  |  |  |  |  |  |  |  |
| Zhu & Cimino [34] | Medical residents from Dept of Medicine at New York Presbyterian Hospital who had access to WebCIS | Computer assisted medication summary of EHR | Pre-post | 6 | Qualitative and quantitative | Not discussed | Small sample size inhibits external validity. Limited sample at one organization also limits external validity |
|  |  |  |  |  |  |  |  |
| Utidjian et al [35] | Patients at one hospital | Using CDSS to determine when palivizumab (expensive treatment) should be administered (under a complicated condition list). | Cluster-randomized trial | 356 | Not discussed | Not discussed | Participants from one hospital only may limit external validity |
| Semler et al [36] | Patients of medical / surgical ICU of academic, tertiary care | CDS for valuation and treatment of sepsis in ICU | Pragmatic Randomized Trial | 407 | Kaplan-Meier and log rank testing and Cox proportion hazards regression | 80% statistical power | Limited sample at one organization also limits external validity |
| Peiris et al [37] | patients in 60 Australian clinics | Computer-guided quality improvement intervention | Randomized Control Trial | 60 clinics | parallel arm cluster | not discussed | Limited sample in one country, limits the external validity |
| Chow et al [38] | one 1500-bed tertiary hospital in Singapore | CDSS for antibiotics | prospective observational cohort study | 1886 | Multi-level statistic regression | Not observed | Limited sample at one organization also limits external validity |
| Wilson et al [39] | Patients in Australia randomly selected and invitied to participate. Only those who responded participated. | Personalized decision support (Internet) tailored to psychological predictors of participation. | Randomized Control Trial | 3408 | Chi squared | not discussed | Only those who responded to the survey from the survey pool were eligible to participate |
| Loeb et al [40] | Providers at one hospital | Training for stepped-care approach to depression | Mixed methods | 58 | Logistic regression | not discussed | Quality-improvement study at one hospital only, which limits the external validity of the findings |
| Mishuris et al [41] | patients who were part of the 2006-2009 national ambulatory and national hospital ambulatory medical care surveys | CDSS | Retrospective cross-sectional analysis | 900 million clinic visits | Logistic regression | not discussed | Not identified |
| Dexheimer et al [42] | Patients who presented for care in Eds between Oct 2010 and Feb 2011 diagnosed with asthma exacerbation | Computerized asthma management system in pediatric ED | Randomized Control Trial | 1339 | Bayesian Network | not discussed | Not identified |
| Heisler et al [43] | Adults with hemoglobin A1C greater 7.5% in clinic in Detroit, Michigan (U.S.) | Customized, interactive, web-based tablet-computer delivered tools | Randomized Control Trial | 188 |  | Not discussed | Limited sample at one organization also limits external validity |
| Eckman et al [44] | Patients in 29 states of the U.S. | Atrial fibrillation decision support tool: (1) no antithrombotic therapy, (2) aspirin, and (3) oral anticoagulant therapy (warfarin in the base case) for each individual patient | Retrospective cohort design | 1876 | not discussed | not discussed | Limited sample in one country, limits the external validity |
| Zaslansky et al [45] | Patients in 17 countries around the world | PAIN OUT which collects patient-reported outcomes (PROs), provides online feedback about the PROs, and serves as a benchmark of PROs for CDSS | Pilot study | 40,898,  60 hospitals,  17 countries | Factor analysis, Cronbach's Alpha, | not discussed | Only those who responded to the survey from the International Pain Outcome Questionnaire were eligible to participate |
| Lobach et al [46] | Medicaid beneficiaries, 3 nurses, 3 social workers, 3 community-health workers, 1 nutritionalist, and 1 health educator in Durham County, North Caroloina (U.S.) | Three CDS modalities: email messages to care managers, printed reports to clinic administrators, and letters to patients. | Randomized Control Trial | 20,180 | Descriptives, means comparisons, 2-sided alpha (0.01) (*P*<.01) | Not discussed | Limited sample in one state, in one country, limits the external validity |
| Barlow & Krassas [47] | Clinicians in Australia | CDSS in clinics | Survey (retrospective) and qualitative | 39 | Chi squared | Not discussed | Small sample and single location limits the external validity |
| Robbins et al [48] | HIV patients at Mass General | Clinical Decision Support | Randomized Control Trial | 33 providers and 1011 patients | Chi squared | not discussed | Limited sample in one state, in one country, limits the external validity |
| Chen et al [49] | records at one facility | Protégé is a free, open source ontology editor creating a guideline-based CDSS without extra coding | Retrospective test of pt data with new CDSS | 2514 | not discussed | not discussed | Data collected at only one organization which limits external validity |
| Seow et al [50] | Patients in Ontario, Canada | Electronic Patient Reported Outcomes on Cancer Care | Retrospective survey | 2,921 | Means comparison | Not discussed | Sample limited to single location which limits external validity |
